# DYT-*TOR1A* dystonia: an update on pathogenesis and treatment

**DOI:** 10.3389/fnins.2023.1216929

**Published:** 2023-08-10

**Authors:** Yuhang Fan, Zhibo Si, Linlin Wang, Lei Zhang

**Affiliations:** ^1^Department of Neurology, the Second Hospital of Jilin University, Changchun, China; ^2^Department of Ophthalmology, the Second Hospital of Jilin University, Changchun, China; ^3^Department of Ultrasound, China-Japan Union Hospital of Jilin University, Changchun, China

**Keywords:** DYT-*TOR1A* dystonia, TorsinA development, *TOR1A* gene, pathogenesis, diagnosis

## Abstract

DYT-*TOR1A* dystonia is a neurological disorder characterized by involuntary muscle contractions and abnormal movements. It is a severe genetic form of dystonia caused by mutations in the *TOR1A* gene. TorsinA is a member of the AAA + family of adenosine triphosphatases (ATPases) involved in a variety of cellular functions, including protein folding, lipid metabolism, cytoskeletal organization, and nucleocytoskeletal coupling. Almost all patients with *TOR1A*-related dystonia harbor the same mutation, an in-frame GAG deletion (ΔGAG) in the last of its 5 exons. This recurrent variant results in the deletion of one of two tandem glutamic acid residues (i.e., E302/303) in a protein named torsinA [torsinA(△E)]. Although the mutation is hereditary, not all carriers will develop DYT-*TOR1A* dystonia, indicating the involvement of other factors in the disease process. The current understanding of the pathophysiology of DYT-*TOR1A* dystonia involves multiple factors, including abnormal protein folding, signaling between neurons and glial cells, and dysfunction of the protein quality control system. As there are currently no curative treatments for DYT-*TOR1A* dystonia, progress in research provides insight into its pathogenesis, leading to potential therapeutic and preventative strategies. This review summarizes the latest research advances in the pathogenesis, diagnosis, and treatment of DYT-*TOR1A* dystonia.

## 1. Introduction

In the 1970s, dystonia was considered a mental illness. As our understanding of these disorders deepened, they are now considered neurological disorders. A milestone in this process was the discovery of the gene *TOR1A* located on human chromosome 9q34 (Ozelius et al., 1997). The gene codes for a protein called “TorsinA,” an adenosine triphosphatase. TorsinA protein is involved in a variety of cellular fuctions, including protein folding, lipid metabolism, cytoskeletal organization, and nuclear polarity (Ozelius et al., 1997; Albanese et al., 2013). The most common mutation in TOR1A is caused by a GAG deletion in the fifth exon of the *TOR1A* gene, resulting in the loss of two adjacent glutamic acid residues (E302) or E303) of TorsinA (“ΔE”-TorsinA), resulting in TorsinA lack of functionality (Ozelius et al., 1997; Charlesworth et al., 2013). ΔE-TorsinA mutation leads to DYT-*TOR1A* dystonia, which is an autosomal dominant disease with a penetrance of only about 30% (Opal et al., 2002). DYT-*TOR1A* is a relatively rare neurological movement disorder, with a global incidence of approximately 1–2 individuals per 100,000 population. However, in certain specific populations, such as Ashkenazi Jews, the incidence of DYT-*TOR1A* can be as high as 100 per 100,000 population, owing to the higher carrier rate of the gene mutation for DYT-*TOR1A* in this population (Ozelius et al., 1997; Saunders-Pullman et al., 2007; Charlesworth et al., 2012). Its main features are involuntary twisting movements and movement disorders that begin in childhood, usually starting in the limbs and gradually spreading to the neck and face muscles, and progressing to severe generalized dystonia. These involuntary movements can significantly impact daily life and mobility, leading to limb stiffness and disability (Albanese et al., 2013). The natural history of DYT-*TOR1A* dystonia suggests a period of vulnerability, as onset occurs in childhood, and carriers of the mutant gene who are asymptomatic in the first 20 years of life often remain asymptomatic throughout life, suggesting that there are other factors that influence the development. Genetic and environmental factors may play in penetrance, study finds (Albanese et al., 2011; Martino et al., 2013). There is currently no cure for the disease, but further research into the pathogenesis of dystonia will hopefully lead to more effective methods for its treatment and prevention. In recent years, research on DYT-*TOR1A* dystonia ha progressed rapidly. Based on the latest research progress, this article reviews the disease characteristics, anatomical basis, pathogenesis, and treatment of DYT-*TOR1A* dystonia.

## 2. Diagnosis and characteristics of DYT-*TOR1A* dystonia

Currently, there are no specific diagnostic criteria for DYT-*TOR1A* dystonia, and diagnosis usually requires considering multiple pieces of information, including (a) medical history: detailed information on the patient’s symptoms, onset time, affected body parts, and disease progression; (b) neurological examination: evaluation of movements and postures, including quantitative assessment of muscle tone and strength; (c) family history: understanding whether other family members of the patient have dystonia or other neurological diseases to help determine whether there is a genetic risk; (d) neuroimaging examination: including magnetic resonance imaging (MRI) and computed tomography to evaluate the brain structure and function of the patient; and (e) genetic testing: testing the patient for *TOR1A* gene mutations. If the genetic mutation result is positive, further disease evaluation is required to confirm the diagnosis (Albanese et al., 2013; Jinnah et al., 2018). It is worth noting that diagnosing DYT-*TOR1A* dystonia requires considering multiple pieces of information, as its clinical presentation overlaps with other dystonias and neurological disorders. Diagnosis based solely on genetic testing is insufficient. DYT-*TOR1A* dystonia is a neurologic movement disorder characterized by progressive muscle tone abnormalities and motor dysfunction, with the following features:

(a) Muscle tone abnormalities: patients experience muscle stiffness and spasms, particularly in the hands, trunk, and neck, resulting in rigid and inflexible limbs or body parts.
(b) Motor dysfunction: patients often exhibit incoordination and abnormal and purposeless movements, particularly in completing fine, complex finger movements. For example, fingers may contort into strange shapes, making simple daily tasks difficult.
(c) Age of onset: DYT-*TOR1A* dystonia typically manifests between the ages of 10 and 30 years; however, a minority of patients may develop it in childhood or middle age.
(d) Genetic features: the disease is typically inherited, caused by a mutation in the *TOR1A* gene.
(e) Slow symptom progression: symptoms of DYT-*TOR1A* dystonia generally progress slowly, but typically do not affect patients’ intelligence or lifespan.
(f) Limited response to treatment: treatment currently focuses on symptom relief.

With limited response to drug treatment, surgery may be a possible option. Additionally, recent studies have shown that DYT-*TOR1A* dystonia is not limited to motor symptoms but may also involve other functional impairments in the nervous system, such as motor learning, memory, and attention (Jahanshahi and Torkamani, 2017). These new findings help to comprehensively understand the characteristics and clinical manifestations of the disease for better diagnosis and treatment.

## 3. Anatomy and neuronal network

DYT-*TOR1A* is a movement disorder involving multiple brain regions at various anatomical levels. Studies have shown that the pathological changes in DYT-*TOR1A* mainly occur in several brain regions, including the cerebral cortex, basal ganglia, and cerebellum.

### 3.1. Cerebral cortex

The cerebral cortex is the starting point of neuronal signals, and its excitability regulation is closely related to motor control. Patients with DYT-*TOR1A* dystonia often have abnormal neuronal activity in the cerebral cortex, especially with symptoms related to dystonia, such as muscle stiffness, tremors, and involuntary movements (Augood et al., 1998). In addition, there are problems in signal transmission and integration between the patient’s brain and basal ganglia, which may affect the regulation of muscle tension (Martella et al., 2009). Furthermore, recent research has found that the interaction between the cerebral cortex and glial cells also plays an essential role in DYT-*TOR1A* dystonia. Glial cells are auxiliary cells of neurons that participate in various physiological functions such as neuron growth, maintenance, and repair (Zhao et al., 2008; Dominguez Gonzalez et al., 2018). In a genetic model of recessive *TOR1A* disease caused by mutations that ablate gene function, Dominguez Gonzalez et al. (2018) found that approximately 30% of *TOR1A* knockout mice exhibited morphological abnormalities in brain development (Cascalho et al., 2020). This work also identifies radial glial cell dysfunction as the explanation for abnormal *Tor1a*-/- brain morphogenesis. The evidence supporting a defect in radial glial cells in *TOR1A* Knock-out mice comes from several observations, including that the proliferative zone of this mice contains mislocalized mitotic nuclei, abnormal cytoarchitecture (Dominguez Gonzalez et al., 2018). Previous studies have also proved that ooc-5, also impairs cell polarity with preventing asymmetric cell division in the early embryo (Basham and Rose, 1999). These evidences have shown that the lack of TorsinA may affect the normal signal transmission and integration between the cerebral cortex and other brain areas by interfering with the polarity of glial cells, resulting in neural network dysfunction (Yokoi et al., 2008; Poston and Eidelberg, 2012; Berryman et al., 2023).

### 3.2. Thalamus

The thalamus is located above the pituitary gland and below the basal ganglia. It comprises multiple subnuclei, including the ventral intermediate nucleus, ventralis oralis anterior and posterior nuclei, centralis medianus nucleus, medial pulvinar nucleus, ventral lateral nucleus, and others. These subnuclei are closely related to the pathogenesis of dystonia. Research has shown that the ventral intermediate nucleus of the thalamus is one of the commonly used targets for deep brain stimulation (DBS) in the treatment of dystonia (Tisch and Kumar, 2020). In addition, abnormal functional connections between the thalamus and cortex are an important anatomical basis for dystonia. Recent studies have also found that subregions of the thalamus, such as the paraventricular nucleus and posterior paraventricular nucleus, may also be involved in the pathogenesis of DYT-*TOR1A* dystonia (Sciamanna et al., 2012b; DeSimone et al., 2016, 2017).

### 3.3. Cerebellum

Based on studies of patients with DYT*-TOR1A* dystonia, the cerebellum is also believed to play an important role in this disease (Carbon et al., 2008). Some studies suggest that the morphology and structure of the cerebellum in patients with DYT-*TOR1A* dystonia differ from those of healthy individuals (Song et al., 2014). For example, MRI studies have found abnormal connections between the cerebellum and cerebral cortex in patients with DYT-*TOR1A* dystonia, as well as changes in connections between the cerebellum and hypothalamus. In addition, some animal studies have found abnormal cerebellar synaptic plasticity in DYT-*TOR1A* mice, suggesting that the cerebellum may be involved in the pathogenesis of DYT-*TOR1A* dystonia (Mazere et al., 2021; Wilkes et al., 2023). Furthermore, the cerebellum may control tics in patients with DYT-*TOR1A* dystonia. One study found that changes in cerebellar cortical activity correlated with the severity of tics in patients with DYT-*TOR1A* dystonia. Another study using functional MRI found that cerebellar cortical activity increased when tics worsened in patients with DYT-*TOR1A* dystonia, suggesting that the cerebellar cortex may play an important role in suppressing tics. Future research is needed to further explore the specific role of the cerebellum in the pathogenesis of DYT-*TOR1A* dystonia to better understand its development and provide more effective treatments (Simonati et al., 1997; Alarcon et al., 2001; Jinnah and Hess, 2006; Neychev et al., 2008; Carbon et al., 2010; Filip et al., 2013; Prudente et al., 2014). By utilizing acute knockdown in adult rodents, Fremont et al. (2017) identified that disruption of TorsinA produces aberrant firing in the cerebellum and results in dystonic symptoms. *In vivo* extracellular recordings were performed on TorsinA KD animals and showed that cerebellar output neurons exhibited erratic burst firing in the presence of TorsinA knockdown in the cerebellum of dystonic animals (Fremont et al., 2017). In a mouse model of the inherited dystonia Rapid-onset Dystonia Parkinsonism (RDP), high-frequency burst firing was shown to underlie severe dystonia (Fremont et al., 2015). Data from Fremont et al. (2015) can demonstrate that dystonia in torsinA KD mice is caused by abnormal and erratic cerebellar output.

### 3.4. Basal ganglia

The relationship between DYT-*TOR1A* dystonia and the basal ganglia has been extensively studied. The basal ganglia is a group of nuclei located deep in the brain, including the striatum, globus pallidus, and substantia nigra, that plays an important role in regulating motor control and higher cognitive functions such as learning and memory. Initially, it was widely believed that dystonia was caused by damage to the basal ganglia, based on the classical model of the basal ganglia circuitry, in which cortical inputs to the striatum are transmitted through the indirect and direct pathways to two main output nuclei, the internal globus pallidus (GPi) and substantia nigra pars reticulata (SNpr) (Ozelius et al., 1997). In this model, the activity of the direct pathway promotes movement by reducing inhibitory output from the GPi, whereas activation of the indirect pathway increases inhibitory output and decreases movement. Dystonia may be caused by an imbalance between the direct and indirect pathways, which leads to abnormally low discharge rates of inhibitory GPi and SNpr neurons that project to the thalamus, thereby reducing inhibition on the thalamus and increasing the excitability of the motor cortex (Coubes et al., 2000). Correspondingly, there is a significant decrease in the discharge rate of GPi neurons after DBS in patients with dystonia. Additionally, an MRI study found that the basal ganglia and brainstem volumes were significantly enlarged in patients with DYT-*TOR1A* dystonia compared with healthy individuals (Coubes et al., 2000). Furthermore, another histological study found neuronal degeneration and glial cell reaction in the basal ganglia and brainstem of patients with DYT-*TOR1A* dystonia. Further anatomical research on DYT-*TOR1A* dystonia is needed to elucidate the pathogenesis of this disorder (Neumann et al., 2017; Liu et al., 2020). Moreover, another neuropathological study found that patients with DYT-*TOR1A* dystonia exhibited neuronal degeneration and glial cell reaction in the basal ganglia and brainstem (Niethammer et al., 2011).

### 3.5. Neuronal network circuitry malfunction

According to recent research, DYT-*TOR1A* dystonia is a network circuitry disorder involving the basal ganglia-cerebellum-thalamus-cortex circuitry (Jinnah et al., 2017). Anatomical and functional imaging-related data on dystonia also provide credible evidence for the concept of dystonia as a network disorder. Differences in the volume of the basal ganglia, cerebellum, and cortex have been observed in patients with idiopathic and inherited dystonia using voxel-based morphometry. However, there are still conflicting reports on the differences in the volume of these regions in patients with DYT-*TOR1A* dystonia compared with healthy individuals (Egger et al., 2007; Pantano et al., 2011). Subsequently, researchers used 18F-fluorodeoxyglucose positron emission tomography to study resting metabolic activity and have identified patterns of regions, including the posterior putamen, pallidum, cerebellum, and supplementary motor area, that explain differences in brain activity between patients (Poston and Eidelberg, 2012). In other words, DYT-*TOR1A* dystonia is not likely caused by dysfunction in a single location but rather a network circuitry disorder. Diffusion tensor imaging studies have also supported this notion, as patients with *TOR1A* dystonia show reduced projection integrity in the cerebellar-thalamo-cortical pathway compared with healthy individuals (Argyelan et al., 2009).

## 4. Mechanism of disease pathogenesis

The pathogenesis of DYT-*TOR1A* dystonia remains controversial and unclear; however, several possible mechanisms exist that are widely accepted, including molecular biology abnormalities of TorsinA, neuronal activity abnormalities, synaptic dysfunction, and environmental factors. Further research is needed in the future to understand the pathogenesis of DYT-*TOR1A* dystonia to develop more effective treatments.

### 4.1. Structure and molecular biology of TorsinA

#### 4.1.1. Structure

**FIGURE 1:**
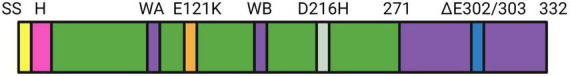
The domain structure of *TOR1A* is shown, which includes the following components: SS signal sequence, H hydrophobic domain, ΔE302/303 (deletion of glutamic acid within the box leading to DYT-TOR1A dystonia), and the transmembrane domain. D216H is a mutation in TorsinA where aspartic acid (D) at position 216 is replaced by histidine (H), which may also affect the susceptibility to DYT-TOR1A dystonia (Herzfeld et al., 2011). E121K is a mutation in the *TOR1A* gene where glutamic acid (E) is replaced by lysine (K). The E121K mutation may lead to changes in the stability, activity, and protein-protein interaction of the TorsinA protein, which can potentially impact its normal cellular function and contribute to the pathogenesis of dystonia.

TorsinA is a AAA + ATPase with 332 amino acids belonging to the family of ATPase (AAA+) proteins associated with various cellular activities, enabling ATP binding and hydrolysis (Vander Heyden et al., 2011). AAA + ATPase utilizes the energy of ATP hydrolysis to unfold or cause conformational changes in substrate proteins. ATP hydrolysis sites include arginine fingers and magnesium ions, and ATP binding sites include nucleotide recognition loops (Walker A motifs) and phosphate esters loop (Walker B motif). Walker A is a conserved lysine residue that contributes to ATP binding; Walker B is a conserved glutamic acid residue that contributes to ATP hydrolysis (Figure 1; Kustedjo et al., 2000, 2003; Hanson and Whiteheart, 2005; Callan et al., 2007; Zhu et al., 2008, 2010; Nagy et al., 2009; Vander Heyden et al., 2011; Zhao et al., 2013). However, TorsinA lacks the arginine fingers necessary to hydrolyze ATP, so TorsinA requires the AAA + -like protein LAP1 or LULL1 to provide arginine fingers to the TorsinA active site, thereby promoting the ATP hydrolysis of TorsinA (Zhao et al., 2013; Brown et al., 2014; Demircioglu et al., 2016). TorsinA lacks the arginine fingers necessary for the hydrolysis of ATP, so TorsinA requires the cofactor lamina-associated polypeptide 1(LAP1) or luminal domain-like LAP1(LULL1) to provide arginine fingers to the TorsinA active site, thereby promoting the ATP hydrolysis of TorsinA. LAP1 and LULL1 are type II transmembrane proteins located in the nuclear membrane and endoplasmic reticulum (Goodchild and Dauer, 2005; Zhao et al., 2013). Most AAA + ATPases assemble into homohexameric structures, but the oligomeric state of TorsinA remains controversial. It was previously suggested that TorsinA forms homohexamers or heterohexamers with LULL1/LAP1 (Zhao et al., 2013; Brown et al., 2014; Demircioglu et al., 2016). Recently, Thomas Schwartz presented a non-canonical filamentous structure. In this structure, TorsinA forms a hollow helical polymer with a period of 8.5 subunits per revolution. These filaments have a broad inner channel with a diameter of about 4 nm. And suggest that TorsinA may bind membrane phospholipids in its hollow interior. And engages and remodels new mechanisms of membrane substrates (Demircioglu et al., 2019).

#### 4.1.2. Subcellular localization and processing

TorsinA is initially synthesized in a non-active form, containing a signal sequence that transports the protein to the ER during synthesis. Subsequently, the signal sequence is cleaved and the protein is further processed and folded, forming an active TorsinA protein (Hewett et al., 2000; Liu et al., 2003; Vander Heyden et al., 2011). TorsinA is mainly localized to the ER. Similar to wild-type (WT) TorsinA, the disease-associated ΔE form is imported into the ER lumen but redistributes to the perinuclear space adjacent to the ER lumen (Gonzalez-Alegre and Paulson, 2004; Goodchild and Dauer, 2004; Naismith et al., 2004). The structure of the NE in eukaryotic cells is conserved, with the inner nuclear membrane (INM) and outer nuclear membrane (ONM) separated by a narrow perinuclear space. Overexpression of TorsinA (ΔE) leads to abnormal INM protrusions into the perinuclear space, resulting in the formation of NE-derived hernias (Gonzalez-Alegre and Paulson, 2004; Goodchild and Dauer, 2004, 2005; Naismith et al., 2004; Grundmann et al., 2012), and a protrusion called “blebs,” which contain ubiquitin and nuclear pore proteins (Liang et al., 2014; Laudermilch et al., 2016). In the highly oxidizing ER environment, the redox state of conserved cysteine residues may regulate TorsinA function (Zacchi et al., 2017). Overexpression of protein disulfide isomerase, which catalyzes the formation of disulfide bonds in ER proteins, reduces the levels of TorsinA, demonstrating that the state of these cysteine residues is a key regulator of TorsinA stability. In addition, ΔE-TorsinA forms abnormal disulfide bond-dependent dimers in overexpression systems, leading to nuclear morphological and functional abnormalities (Hettich et al., 2014; Vulinovic et al., 2014). A recent experiment simulating DYT-*TOR1A* using patient-specific cholinergic motor neurons (MNs) generated from either transformed patient skin fibroblasts or induced pluripotent stem cells showed that human MNs with heterozygous *TOR1A* mutations display reduced neurite length and branching, markedly thickened NEs, disrupted nuclear shape, and impaired nuclear-cytoplasmic transport of mRNA and proteins, but lack the “blebs” frequently observed in animal models. It was also found that Lamin B1(LMNB1) is upregulated in DYT-*TOR1A* cells and exhibits abnormal subcellular distribution, specifically in cholinergic MNs. Lamins are V-type intermediate filament proteins located below the inner nuclear membrane, where they form a highly ordered network called nuclear lamina (Burke and Stewart, 2013; Xie and Burke, 2016). The nuclear lamina can contribute to nucleus shape and mechanical stability, as well as regulating chromatin organization. In mammals, Lamins are divided into Lamin A and Lamin B. Among them, overexpression of LMNB1 has been shown to increase nuclear rigidity (Wintner et al., 2020). Interestingly, LMNB1 downregulation greatly improves the NE morphology and nuclear transport defects in DYT-*TOR1A*. This suggesting that LMNB1 dysregulation may constitute a major molecular mechanism of DYT-*TOR1A* pathology and provide a new molecular target for intervention (Ding et al., 2021). Although there is some understanding of TorsinA’s subcellular localization and processing, its detailed mechanism still needs further study to deepen our understanding of its function and relationship with DYT-*TOR1A* dystonia. To date, the degradation mechanism of TorsinA remains controversial. Some studies suggest that TorsinA may be degraded through the proteasome pathway, whereas others suggest that TorsinA may be degraded through the autophagy pathway (Giles et al., 2008; Gordon and Gonzalez-Alegre, 2008). A recent study suggested that the degradation mechanism of ΔE-TorsinA may involve abnormalities in the nuclear-cytoplasmic transport (NCT). Specifically, ΔE-TorsinA may interfere with the assembly of NPC in interphase cells, thereby affecting the normal process of NCT (Ding et al., 2021). These findings provide new insights into the degradation mechanism of TorsinA, and future studies may further explore these mechanisms and their role in DYT-*TOR1A* dystonia (Gordon et al., 2012; Kwon et al., 2013; Brown et al., 2014). The steady-state level of △E-TorsinA is lower than that of WT TorsinA, indicating that TorsinA (ΔE) may have a dominant negative effect on the WT protein (Giles et al., 2008; Gordon and Gonzalez-Alegre, 2008). Therefore, the ratio of TorsinA(△E) to TorsinA(WT) expression is important in the pathogenesis of DYT-*TOR1A* dystonia. In the future, further research will explore the interactions between the oxidative/reductive state, oligomerization, stability, and function of TorsinA.

### 4.2. Cellular neurobiology

Evaluating how TorsinA expression in the ER and NE affects cellular physiological functions is a reasonable first step in identifying downstream biological events relevant to TorsinA function. Expanding the focus from organelle biology to other cellular mechanisms affected by TorsinA function can help fill gaps in our understanding of DYT-*TOR1A* gene defects and systems neuroscience. While many questions remain unanswered, significant research progress has been made to date.

#### 4.2.1. Aberrant ATPase activation

**FIGURE 2:**
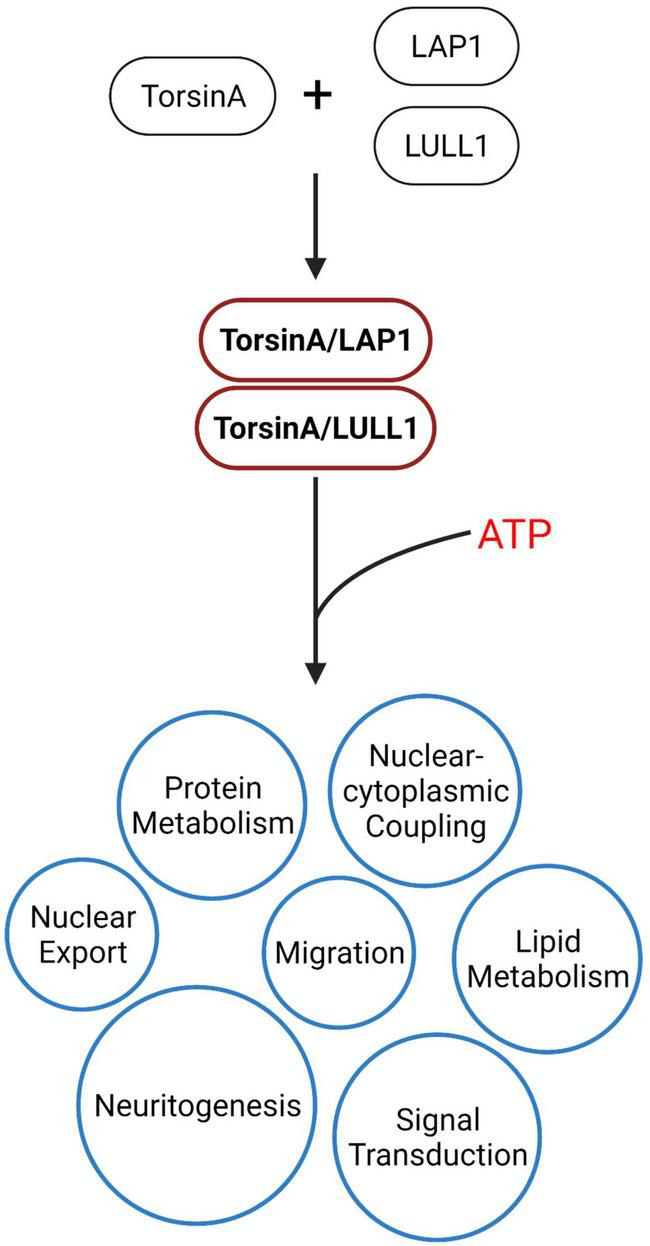
TorsinA is an AAA + ATPase, but unlike other AAA + proteins, TorsinA lacks the conserved catalytic arginine residue. Studies have shown that TorsinA is completely inactive when isolated and requires LAP1/LULL1 to provide arginine residues, which are necessary for TorsinA to strongly induce ATP hydrolysis (Zhao et al., 2013). The energy generated from this ATP hydrolysis is involved in downstream biological processes, including ribosomal export, protein metabolism, nucleo-cytoplasmic coupling, lipid metabolism, cell migration, and signal transduction, which play critical roles in maintaining normal cellular functions and biological processes.

TorsinA is a member of the AAA + (ATPases associated with various cellula activities) superfamily. However, unlike typical ATPases, TorsinA has a complete AAA + domain but lacks the arginine fingers necessary for hydrolysis of ATP, so TorsinA does not have ATPase activity (Ogura et al., 2004; Kock et al., 2006). TorsinA requires LAP1 and LULL1 as cofactors to bind to it and provide it with an arginine finger to hydrolyze ATP (Zhao et al., 2013; Brown et al., 2014; Rose et al., 2015; Laudermilch and Schlieker, 2016; Chase et al., 2017). In patients with DYT-*TOR1A* dystonia, mutations in the *TOR1A* gene impairs the binding of TorsinA to LAP1/LULL1, thereby compromising the ability of TorsinA to hydrolyze ATP (Zhao et al., 2013; Sosa et al., 2014; Demircioglu et al., 2016). Furthermore, lap1-deficient mice exhibited early perinatal death, further emphasizing that dysfunctional TorsinA-ATPase activation may play a critical role in the pathogenesis of DYT-*TOR1A* dystonia (Kim et al., 2010). TorsinA utilizes the ability generated by ATP hydrolysis to participate in various biological processes such as membrane transport, cytoskeleton dynamics, vesicle fusion and NPC assembly in interphase cells (Vale, 2000; Hanson and Whiteheart, 2005; Granata et al., 2009). Understanding the effect of ΔE-TorsinA on ATPase activation is a critical step toward understanding the pathogenesis of DYT-*TOR1A* (Figure 2).

#### 4.2.2. Mislocalization of △E-TorsinA

TorsinA is located on the ER (endoplasmic reticulum) and NE (nuclear envelope) cell membranes and participates in important biological processes such as stabilizing nuclear morphology, cell division, and regulating nuclear membrane morphology by interacting with other proteins. The nuclear membrane is a double-layered membrane composed of the INM (inner nuclear membranes) and ONM (outer nuclear membranes), separated by a narrow perinuclear space, which mainly functions to separate the cell nucleus and cytoplasm and to regulate the transport of substances between the nucleus and cytoplasm (Goodchild and Dauer, 2004, 2005). Overexpression of TorsinA(ΔE) can lead to abnormal protrusions of the INM into the perinuclear space, forming blebs, and causing the formation of inclusion bodies derived from the NE. This defect in the NE structure can be observed by manipulating TorsinA in other model systems, including humans, flies, and worms (Hewett et al., 2000; Breakefield et al., 2001; Gonzalez-Alegre and Paulson, 2004; Goodchild and Dauer, 2004, 2005; Naismith et al., 2004; Goodchild et al., 2005; Grundmann et al., 2012; Jokhi et al., 2013; VanGompel et al., 2015; Laudermilch et al., 2016). The blebs contain ubiquitin and nucleoporin (Liang et al., 2014; Laudermilch et al., 2016). Consistent with this, previous studies have found two minor changes between the brains of patients with DYT-*TOR1A* dystonia and controls: cell body enlargement of dopamine neurons (Rostasy et al., 2003) and ubiquitin-containing inclusion bodies in the midbrain (McNaught et al., 2004). In conclusion, the abnormal localization of ΔE-TorsinA leading to nuclear membrane dysfunction is one of the important causes of DYT-*TOR1A* dystonia.

#### 4.2.3. Disruption of ER function

The ER is a complex network within the cell with functions including protein synthesis, transport and modification of membrane proteins, lipid metabolism, calcium ion balance, and quality control. When ER function is disrupted, these biological processes are affected, impairing cell function (Torres et al., 2004; Hewett et al., 2007; Burdette et al., 2010; Chen et al., 2010; Nery et al., 2011; Kakazu et al., 2012; VanGompel et al., 2015; Beauvais et al., 2016; Grillet et al., 2016; Saunders et al., 2017; Dominguez Gonzalez et al., 2018; Pappas et al., 2018a). The function of TorsinA in the ER and the role of ΔE-TorsinA in DYT-*TOR1A* dystonia are not fully understood. In one study, fibroblasts from patients with DYT-*TOR1A* dystonia and cultured neurons from TorsinA-null mice show impaired ER processing of a secreted reporter protein found under homeostatic conditions (Hewett et al., 2007). Another study indicated that fibroblasts and neurons from TorsinA-null mice and homozygous ΔE-TorsinA knock-in mice exhibited increased sensitivity to ER stress (Cho et al., 2014). These studies suggest that ΔE-TorsinA may cause ER dysfunction by interfering with ER protein metabolism.

#### 4.2.4. Protein folding and quality control mechanisms

**FIGURE 3:**
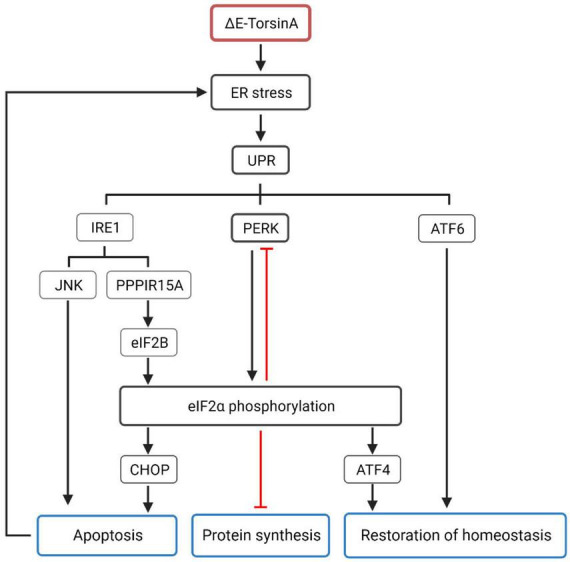
Mutated TorsinA, specifically the ΔE-TorsinA mutant, can activate the unfolded protein response (UPR), which is a major cellular response to endoplasmic reticulum (ER) stress (Kim et al., 2015). UPR consists of three main signaling pathways: IRE1, PERK, and ATF6. The PERK-eIF2α pathway is the most important pathway in which activated PERK phosphorylates eIF2α, leading to inhibition of global protein synthesis. Additionally, there is a feedback mechanism between eIF2α and PERK, where phosphorylated eIF2α can inhibit the synthesis of PERK itself during ER stress, maintaining the balance of ER stress response and preventing excessive stress-induced damage to cells (Beauvais et al., 2018). The IRE1 pathway can induce cell apoptosis by activating the JNK signaling pathway and inhibiting the activity of eIF2B through the activation of PPP1R15A, resulting in increased phosphorylation of eIF2α and affecting the level of protein synthesis (Beauvais et al., 2019). ATF6 is an important member of the UPR and belongs to the transcription factor family. It is activated during ER stress and participates in the transcriptional regulation of a series of genes related to protein synthesis and repair. The phosphorylation status of eIF2α also triggers a series of cellular adaptive responses. For example, phosphorylated eIF2α increases the translation of ATF4 mRNA, leading to the synthesis of ATF4 protein. ATF4, together with ATF6, promotes the restoration of ER homeostasis. When ER stress worsens, the phosphorylation level of eIF2α further increases, leading to increased expression of CHOP, which exacerbates the process of cell death and further damages ER stress (Pappas et al., 2018b).

TorsinA exhibits chaperone activity *in vitro* (Burdette et al., 2010) and assists substrates in folding into their native conformations. It also coordinates material transport between the ER and the Golgi apparatus (Hewett et al., 2007, 2008; Burdette et al., 2010), and affects the degradation of misfolded ER cargo through ER-associated degradation (Nery et al., 2011). In addition, worm experiments support the role of TorsinA in maintaining protein homeostasis in the ER, indicating that TorsinA is a modulator of the ER stress response (Chen et al., 2010). Overexpression of TorsinA appears to protect cells from ER stress, whereas downregulation of TorsinA sensitizes cells to ER stress (Nery et al., 2011). Under steady-state conditions, when the level of misfolded proteins in the ER lumen reaches a threshold, a carefully orchestrated adaptive program called the unfolded protein response (UPR) is activated to restore ER homeostasis (Bellato and Hajj, 2016; Hetz and Saxena, 2017). EIF2α is a key effector of the UPR, and UPR activation results in the phosphorylation of eIF2α by the ER-specific kinase PERK. Specifically, during ER stress, viral infection, or inflammation, cells utilize their eIF2α kinases to suppress the translation of unnecessary proteins (Dabo and Meurs, 2012; Bond et al., 2020), by inhibiting translation to reduce synthetic metabolic burden while allowing for the synthesis of a set of proteins to restore homeostasis, such as transcription factor ATF4. There is a feedback mechanism between eIF2α and PERK in response to ER stress. When eIF2α is phosphorylated under ER stress conditions, it can inhibit the synthesis of PERK itself, which regulates the activity of PERK and maintains the balance of ER stress response, preventing excessive stress response from damaging the cell. Activation of the IRE1 (endoplasmic reticulum-associated kinase 1) signaling pathway can induce apoptosis in cells by activating the JNK (c-Jun N-terminal kinase) signaling pathway. In addition, IRE1 can also inhibit the activity of eIF2B (eukaryotic translation initiation factor 2B) by activating PPP1R15A (protein phosphatase 1 regulatory subunit 15A), leading to an increase in the phosphorylation status of eIF2α, thereby affecting the level of protein synthesis. ATF6 (activating transcription factor 6), a transcription factor that is also involved in the UPR, is also activated during ER stress and regulates the transcription of a series of genes related to protein synthesis and repair, promoting cell adaptation to stress. When ER stress is sustained, CHOP (CCAAT-enhancer-binding protein homologous protein) expression is upregulated, which may ultimately lead to caspase 3 activation and cell apoptosis (Figure 3; Baptista et al., 2003; Hewett et al., 2003; Gordon et al., 2011; Cho et al., 2014; Beauvais et al., 2016, 2018, 2019; Rittiner et al., 2016). In addition, enhancing the eIF2α signal can alleviate the lethality caused by homozygous torsinA ΔE in DYT-*TOR1A* mice (Beauvais et al., 2016, 2019). Aberrant regulation of the eIF2α signaling pathway and therapeutic manipulation of eIF2α phosphorylation further support the notion that eIF2α signaling pathway dysregulation is one of the pathogenic mechanisms underlying DYT-*TOR1A* dystonia. Taken together, ΔE-TorsinA can affect protein folding and quality control mechanisms of the ER through multiple mechanisms, leading to protein aggregation and neuronal damage, ultimately resulting in the development of DYT-*TOR1A* (Shroff et al., 2021).

#### 4.2.5. Lipid metabolism

**FIGURE 4:**
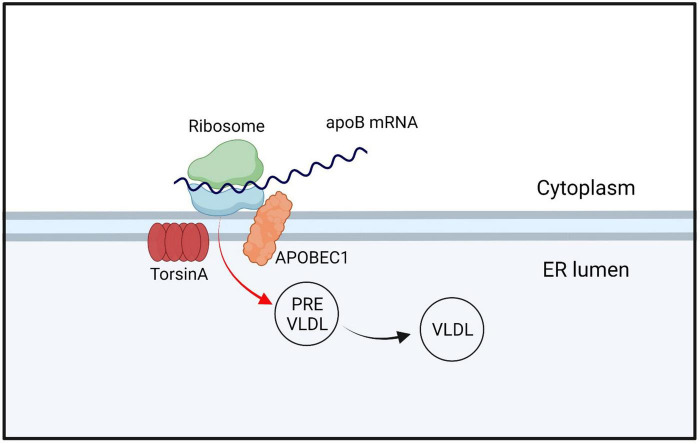
TorsinA/LAP1 interacts with APOBEC1 and regulates the expression and function of apoB100 by editing specific positions on apoB mRNA, which encodes for apoB. After entering the endoplasmic reticulum (ER), apoB undergoes lipidation to form VLDL precursors, which mature and move to the Golgi apparatus when they reach a certain size. During this process, VLDL is packaged into specialized vesicles, which may also involve the participation of TorsinA. The term “apoB mRNA” refers to the mRNA molecule that encodes apoB100, while APOBEC1 stands for Apolipoprotein B mRNA editing enzyme, catalytic polypeptide 1.

**FIGURE 5:**
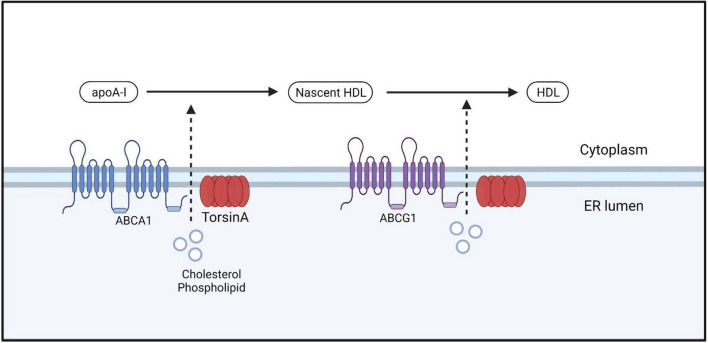
ABCA1 facilitates the efflux of free cholesterol and phospholipids from the cell membrane by forming a lipoprotein complex, which is then transported out of the cell. This lipoprotein complex is accepted by the lipid-binding protein apoA-I in the extracellular space, leading to the formation of nascent HDL. Nascent HDL further accepts free cholesterol and phospholipids bound to phosphatidylcholine that are effluxed by ABCG1, resulting in the maturation of HDL.

**FIGURE 6:**
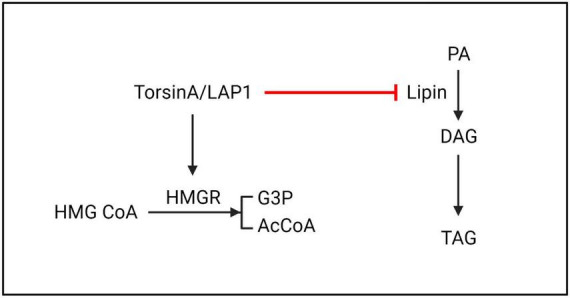
TorsinA/LAP1 may promote the conversion of HMG CoA to G3P and AcCoA by facilitating HMGR. Additionally, lipin enzyme has the ability to catalyze the dephosphorylation of PA to generate DAG. Existing evidence suggests that TorsinA inhibits lipin enzyme through a post-translational mechanism, resulting in further suppression of PA metabolism. HMGR, 3-hydroxy-3-methylglutaryl-CoA reductase; HMG CoA, 3-hydroxy-3-methylglutaryl coenzyme A; G3P, glycerol-3-phosphate; AcCoA, acetyl-CoA; PA, phosphatidic acid; DAG, diacylglycerol; TAG, triacylglycerol.

The ER lipid metabolism network is crucial for cell growth, membrane composition, organelle function, and production of lipid storage molecules for energy (Fagone and Jackowski, 2009; Holthuis and Menon, 2014). It also serves as the foundation for cellular energy storage, and dysfunction in this network can lead to metabolic issues in humans, including malnutrition and diabetes (Huang-Doran et al., 2010). Currently, the relationship between DYT-*TOR1A* dystonia and lipid metabolism is unclear. DYT-*TOR1A* patients exhibit significantly lower baseline serum cholesterol levels relative to normal populations, as well as lower cholesterol content in the liver. However, no symptoms or complications associated with lowered cholesterol levels have been identified (Shin et al., 2019). DYT-*TOR1A* patients exhibit significantly lower baseline serum cholesterol levels relative to normal populations, as well as lower cholesterol content in the liver. However, no symptoms or complications associated with lowered cholesterol levels have been identified (Shin et al., 2019). Studies have found that the TorsinA/LAP1 complex can increase the activity of HMG-CoA reductase, thereby increasing cholesterol synthesis and leading to elevated cholesterol levels (Cascalho et al., 2020). These findings suggest that TorsinA has an inseparable relationship with cholesterol metabolism. However, the exact mechanism of this relationship remains unclear. TorsinA/LAP1 can also regulate the activity of acyl-CoA:cholesterol acyltransferase 1 (ACAT1), which is involved in cholesterol esterification, converting cholesterol to cholesterol esters for storage. TorsinA deficiency can lead to reduced ACAT1 activity, resulting in decreased synthesis and storage of cholesterol esters (Fichtman et al., 2019). In addition, TorsinA plays a significant role in the metabolism of very low-density lipoprotein (VLDLs). Apolipoprotein B100 (apoB100) is a major constituent of VLDLs and the characteristic lipid-carrying protein. After apoB100 is translated into protein, it enters the endoplasmic reticulum (ER) and undergoes lipidation with the assistance of microsomal triglyceride transfer protein (MTP), that is, the addition of phospholipid (PL) and triglyceride (TG) to form the initial Low-density VLDL precursor. As the VLDL precursors grow to a certain size within the ER, they are transported to the Golgi apparatus, where they are subsequently processed into mature VLDL particles. Subsequently, VLDL is packaged into specialized vesicles, a process that may require the participation of TorsinA (Shin et al., 2019; Figure 4). Furthermore, TorsinA also interacts with several other proteins involved in cholesterol transport, including ABCA1 and ABCG1. ABCA1 and ABCG1 are ATP-binding cassette (ABC) transporters located on the cell membrane that play critical roles in the transport of cholesterol and phospholipids. However, they differ in their transport pathways, tissue expression, and biological functions. Apolipoprotein A-I accepts cholesterol effluxed by ABCA1, generating nascent HDL, which subsequently accepts cholesterol effluxed by ABCG1, forming mature HDL. While ABCA1 can directly transport or flip phospholipids across the lipid bilayer; the mechanism by which ABCA1 mediates cholesterol efflux to apolipoprotein A-I is not clear. By modulating the activity of these proteins, TorsinA may regulate intracellular cholesterol transport, affecting its availability for cellular processes, and may contribute to disease development (Bazioti et al., 2022; Figure 5). TorsinA can also controls lipid metabolism by inhibiting the activity of lipin phosphatidic acid phosphatase (PAP) (Grillet et al., 2016). Eukaryotic cells have two types of PAP: lipins act on the ER and depend on magnesium, whereas non-specific lipid phosphatases act on other regions of the cell (Csaki et al., 2013; Jacquemyn et al., 2017; Figure 6). In mammals, lipin enzymes have three isoforms, among which LIPIN1 and LIPIN2 are expressed actively in the mammalian brain (Donkor et al., 2007; Dwyer et al., 2012). Phosphatidic acid and diacylglycerol are precursor lipids in the competitive branch of phospholipid synthesis and signal lipids that regulate several pathways, including Erk and mTOR (Nadra et al., 2008; Csaki et al., 2013; Cascalho et al., 2017). By acting on these enzymes, TorsinA can serve as a switch to direct lipid metabolism toward energy storage or membrane biogenesis (Teleman, 2016; Cascalho et al., 2017) and affect synaptic physiology (Han et al., 2007). Studies have shown that upregulation of lipin PAP activity can prevent axonal regeneration after injury (Yang et al., 2020; Figure 6). Furthermore, it was found that reducing the *LPIN1* gene increased survival in a mouse model of *TOR1A* disease, while inhibiting neurodegeneration, motor dysfunction, and nuclear membrane pathology (Cascalho et al., 2020). These data confirm that *TOR1A* disease mutations cause abnormal phosphatidic acid metabolism, and provide a theoretical basis for treating DYT-*TOR1A* dystonia by inhibiting lipin PAP enzyme activity.

#### 4.2.6. Calcium homeostasis

Calcium is a ubiquitous second messenger crucial for neuronal function and survival, regulating multiple signaling pathways. Calcium homeostasis is maintained by regulating the entry of extracellular calcium through the plasma membrane or releasing calcium from intracellular stores such as the ER (Bollimuntha et al., 2017). Abnormalities in calcium currents, levels, and function of calcium-dependent channels, including ER calcium pumps, L-type calcium channels, TRPC3, and TRPC4, were measured in striatal neurons of the DYT-*TOR1A* knock-in mouse model (Pisani et al., 2006; Sciamanna et al., 2011, 2014; Dang et al., 2012; Iwabuchi et al., 2013a,b; Ponterio et al., 2018). ΔE-TorsinA may interact with these calcium channels and calcium ion-related proteins, thereby affecting the concentration and homeostasis of intracellular calcium ions. However, this does not prove that torsinA directly affects calcium physiology but may more likely reflect upstream defects in neurotransmission. ΔE-TorsinA may also affect intracellular calcium ion homeostasis by regulating synaptic plasticity. Synaptic plasticity is a key mechanism for communication between neurons, including the regulation of calcium ion signaling. Studies have shown that TorsinA(ΔE) may cause the calcium sensor synaptotagmin1 to aggregate on the cell surface by affecting stonin2, which may affect transport (Granata et al., 2011) and serve as a basis for further influencing the physiology of synaptic vesicles (Granata et al., 2008, 2009; Warner et al., 2010; Kakazu et al., 2012). It was found that abnormal calcium physiology observed in DYT-*TOR1A* knock-in cerebellar tissue slices are more pronounced under ER stress conditions (Beauvais et al., 2016), suggesting that abnormal calcium physiology in DYT-*TOR1A* dystonia may be related to ER stress. Further understanding of the relationship between DYT-*TOR1A* dystonia and calcium physiology will provide guidance for understanding the pathogenesis of DYT-*TOR1A* dystonia and exploring calcium defect correction as a potential therapeutic strategy.

#### 4.2.7. Abnormal nucleocytoplasmic transport

**FIGURE 7:**
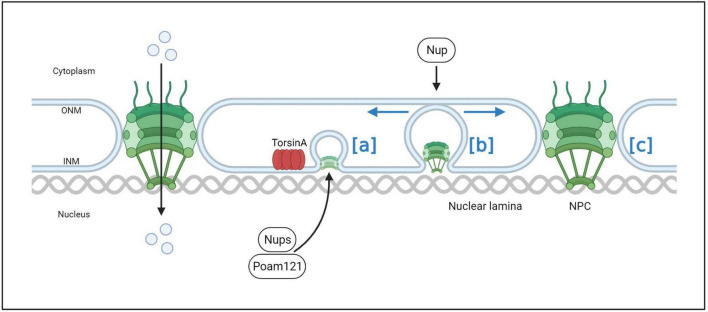
The process of normal NPC assembly. In normal interphase NPC assembly, POM121 is recruited to the inner nuclear membrane, and NPC components and subcomplexes shuttle into mature pores. TorsinA helps facilitate NPC assembly and growth by interacting with POM121 (a). As the NPC intermediate matures, Nups that may induce membrane invagination are added in a process that drives complex growth in the lateral and outward nuclear membrane directions (b). After membrane fusion events, late and cytoplasmic Nups such as Nup358 are added to eventually form a complete NPC (c).

**FIGURE 8:**
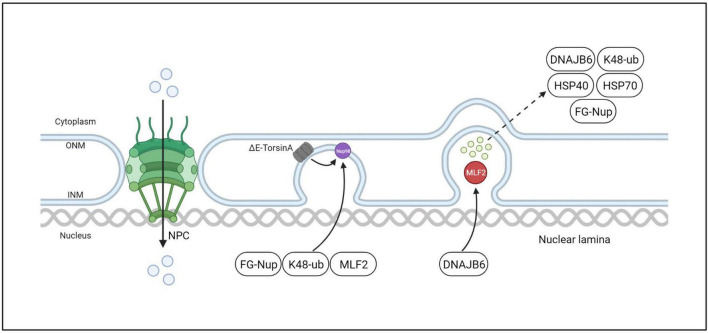
ΔE-TorsinA in-cell, interphase NPC biogenesis model. The mutant ΔE-TorsinA leads to abnormal localization and function of Nup98 in NPCs, resulting in its sequestration into bleb-like structures and recruitment of FG-Nups, K48-ub, MLF2, etc. MLF2 further directs recruitment of DNAJB6, which is a member of the molecular chaperone family. DNAJB6 is believed to be a regulator of protein quality control, interacting with proteins to target them for degradation by the ubiquitin-proteasome system.

The NE is a defining characteristic of eukaryotic cells that separates the nucleoplasm from the cytoplasm. The exchange of information and material between the two compartments is mediated by a large molecular complex embedded in the NE called NPCs. NPCs are complex protein assemblies located on the nuclear membrane that play important biological roles in nucleocytoplasmic transport, ribosome assembly, RNA transport, and other functions. The NPC is composed of nucleoporins, some of which contain intrinsically disordered phenylalanine-glycine domains that form a dense hydrogel and establish the permeability barrier properties of the NPC (Ribbeck and Gorlich, 2001; Frey and Gorlich, 2007; Hülsmann Bastian et al., 2012; Schmidt and Gorlich, 2016). In TorsinA-deficient cells, a group of FG-Nups was observed at the electron density base of NE blebs. The diameter of this density was similar to that of mature NPCs, indicating a possible involvement of TorsinA in the biogenesis of NPCs (Laudermilch et al., 2016; Figure 7). In normal interphase NPC assembly, POM121 is recruited to the inner nuclear membrane, and NPC components and subcomplexes shuttle into mature pores. TorsinA helps facilitate NPC assembly and growth by interacting with POM121 (Figure 7A). As the NPC intermediate matures, Nups that may induce membrane invagination are added in a process that drives complex growth in the lateral and outward nuclear membrane directions (Figure 7B). After membrane fusion events, late and cytoplasmic Nups such as Nup358 are added to eventually form a complete NPC (Figure 7C). As a result of NE blebbing, nuclear transport defects have been observed in Torsin-defective models, including induced pluripotent stem cell-derived neurons from patients (VanGompel et al., 2015; Tanabe et al., 2016; Pappas et al., 2018a). “Blebs” are thought to be intermediate products of abnormal NPC biogenesis that are stalled before INM and ONM fusion (Dorboz et al., 2014; Demircioglu et al., 2019; Fichtman et al., 2019). Although no mature NPCs are found within “blebs,” specific nucleoporins associated with multiple subcomplexes that construct NPCs have been found within these “blebs” (Jungwirth et al., 2010; Dorboz et al., 2014; Kayman-Kurekci et al., 2014; Fichtman et al., 2019). In addition, the diagnostically relevant components of these vesicles, such as the protein myeloid leukemia factor 2 (MLF2) and K48-linked ubiquitin (Ub) chains, are not well-characterized. Prophet et al. (2022) used a biotin-based proximity labeling technique and found that FG-Nup Nup98 is necessary for vesicle formation, suggesting its role in recruiting other FG-Nups during NPC assembly. They also identified K48-Ub and MLF2 in cytoplasmic vesicles, possibly secreted by FG-Nups. The TorsinA complex can interact with Nup98 to regulate its localization and function in NPC (Prophet et al., 2022; Figure 8). Moreover, in contrast to the nuclear transport defects caused by impaired NPC assembly in cells with conventional TorsinA defects reported in the literature, Prophet et al. (2022) also discovered a dual protein toxicity effect of NE blebs, providing a new hypothesis for the pathogenesis of DYT- *TOR1A* dystonia. Overall, these reports suggest that TorsinA affects the biogenesis of NPCs during neuronal maturation, and loss of TorsinA function prevents their evolution through normal developmental processes.

#### 4.2.8. Cellular cytoskeleton dynamics

**FIGURE 9:**
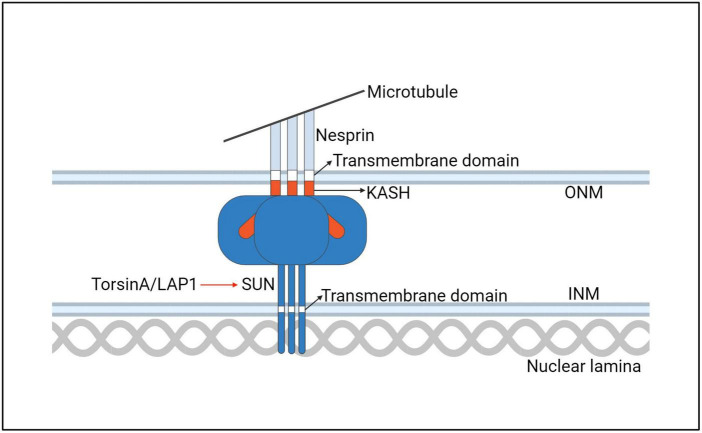
A model for the interaction of the TorsinA with the LINC complex. ONM, outer nuclear membrane; INM, inner nuclear membrane.

**FIGURE 10:**
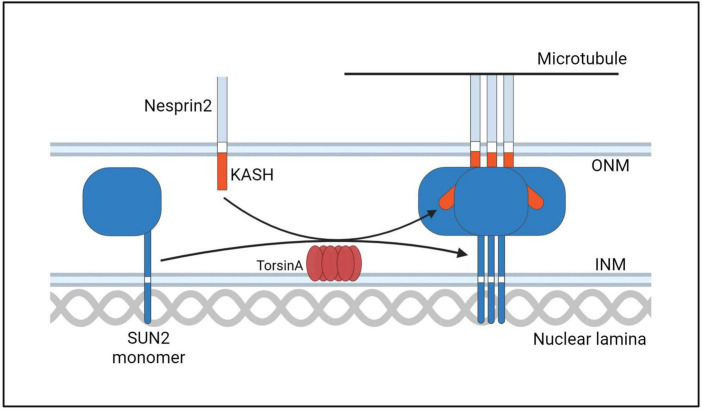
A hypothetical model of TorsinA involvement in LINC complex assembly. TorsinA may mediate the formation of LINC complex in two ways. First, by interacting with KASH, Nesprin2 is promoted to combine with Microtubule. Second, by promoting the formation of trimers from SUN2 protein monomers. Thereby promoting the assembly of LINC (Starr and Rose, 2017).

Mechanotransduction signals play an important role in cell division, differentiation, and migration. The nucleus adjusts according to the mechanical signals it receives. This mechanically driven cell change can not only effectively transmit mechanical signals to the nucleus, but also through the nuclear membrane. Mechanistic signals dynamically adjust the nuclear cytoskeleton structure and maintain genome integrity, thereby affecting downstream biological responses (Chang et al., 2015; Uzer et al., 2016). The INM and ONM are separated by a lumen called the perinuclear space (PNS), but are connected to each other where the nuclear pores insert. Spanning the two membranes and linking the nuclear and cytoskeleton is an important family of protein complexes: the LINC (Linker of Nucleoskeleton and Cytoskeleton) complex. The LINC complex consists of Klarsicht, ANC-1, and Syne homology (KASH) domain proteins on ONM and Sad1 and UNC-84 (SUN) domain proteins on INM (Saunders and Luxton, 2016; Uzer et al., 2016). The LINC complex can transmit mechanical force on the nuclear membrane, which plays an important role in moving the nucleus, maintaining the connection between the centrosome and the nucleus, and signal transduction (Zhang et al., 2009; Fridolfsson and Starr, 2010; Luxton and Gundersen, 2011). SUN proteins have been reported to play a role in the localization of TorsinA to the nuclear membrane, and TorsinA interacts with multiple KASH domains. And a recent study found that loss of *TOR1A* disrupts the localization of nesprin3, the KASH protein that connects the nucleus to the intermediate filament (Saunders and Luxton, 2016; Saunders et al., 2017). Although widely speculated, the relationship between DYT- *TOR1A* dystonia and LINC complex dysfunction remains unclear. However, ΔE-TorsinA disrupts some LINC complex-dependent functions, such as cell migration and polarization (Nery et al., 2008; Vander Heyden et al., 2009; Jungwirth et al., 2011; VanGompel et al., 2015; Saunders and Luxton, 2016; Hennen et al., 2018; Pappas et al., 2018a). ΔE-TorsinA may also affect the dynamic reorganization and structural stability of the cytoskeleton, thus affecting the morphology and structure of the nuclear-cytoskeletal connection (Figure 9). LINC complex is a heterohexamer composed of KASH and SUN proteins, and TorsinA may play a role in its assembly (Figure 10). Although TorsinA gene-knockout mice do not exhibit significant developmental defects in the brain, they are usually associated with impaired neuronal migration; however, subtle changes in neuronal migration of the forebrain and ganglionic eminences of embryos can be observed in the absence of TorsinA (de Anda et al., 2005; Goodchild et al., 2005). In developing hippocampal neurons, the correlation between the position of the centrosome and axon initiation has been demonstrated (de Anda et al., 2005). As many cellular components and mechanisms required for axon initiation are similar to those required for centrosome orientation in directed cell migration, TorsinA may also affect the establishment of axon-dendrite polarity in neurons (Li and Gundersen, 2008), which is supported by an experiment on the cerebello-thalamo-cortical pathway of ΔE mutation heterozygous mice using population-wide circuit imaging (Ulug et al., 2011). In a wound healing assay, fibroblasts of ΔE-TorsinA mice exhibited impaired rearward nuclear movement during centrosome orientation and cell migration, possibly due to defects in nuclear movement during centrosome orientation (Saunders et al., 2017). OOC-5, a TorsinA homolog, is lacking in the nematode *C. elegans*, exhibiting defects in cell polarity and asymmetric division (Basham and Rose, 1999, 2001). In summary, further research on the relationship between TorinA and LINC complex may provide a reference for understanding the pathogenesis of DYT-*TOR1A* dystonia. Future research needs to explore the in-depth relationship between ΔE-TorsinA and the nuclear-cytoskeletal connection and how these interactions affect the morphological and mechanical of cells.

### 4.3. Synaptic function and neurodevelopment

Synapses are the junctions between neurons and play a critical role in neural signal transmission. Currently, DYT- *TOR1A* dystonia is widely recognized as a neurodevelopmental disorder, and the relationship between TorsinA and mouse survival, as well as the human neurological phenotypes resulting from its mutation, suggests an important relationship between TorsinA and synaptic function and neurodevelopment (Dang et al., 2005; Goodchild et al., 2005; Kariminejad et al., 2017; Reichert et al., 2017; Isik et al., 2019). In synapses, TorsinA regulates the interaction of membrane proteins at the presynaptic and postsynaptic sites, maintains the structure and function of neurons, and regulates protein transport and synaptic function within neurons. Studies have confirmed the effects of ΔE-TorsinA on synaptic function, including transport defects, impaired synaptic vesicle cycling, synaptic plasticity defects, and axon formation defects (Dang et al., 2005; Dauer, 2014; Liang et al., 2014; Tanabe et al., 2016; Kariminejad et al., 2017; Reichert et al., 2017; Cascalho and Goodchild, 2018; Maltese et al., 2018; Isik et al., 2019). Axon formation defects may be caused by synaptic plasticity defects. Synaptic plasticity is an important concept in the nervous system, referring to the strength and efficiency of synaptic connections between neurons to be adjusted owing to changes in neural activity. This synaptic plasticity is the basis of normal nervous system development and neural behaviors such as learning and memory. Specifically, ΔE-TorsinA may interfere with the structure and transport pathways of the Golgi and nucleolus within neurons, thus affecting the synthesis, assembly, and transport of synaptic proteins, ultimately leading to structural and functional synaptic disorders. Studies have found that ΔE-TorsinA may also cause abnormal regulation of postsynaptic calcium ion concentration, thereby affecting synaptic plasticity. In addition, ΔE-TorsinA may interfere with the eIF2α signal transduction pathway within neurons, thereby affecting synaptic plasticity (Costa-Mattioli et al., 2007; Di Prisco et al., 2014; Moon et al., 2018). In addition to participating in ER stress, eIF2α signaling also participates in regulating downstream synaptic plasticity changes and neuronal extension. These plasticity defects may lead to abnormal neuronal function, thereby causing symptoms of DYT- *TOR1A* dystonia. Experimental data have shown that abnormal synaptic plasticity expressed in DYT-*TOR1A*-knock-in mice can be corrected by regulating the eIF2α signaling pathway (Rittiner et al., 2016). Recently, researchers have modeled DYT- *TOR1A* dystonia using human patient-specific cholinergic motor neurons (MNs), which were generated by direct conversion of patient skin fibroblasts or induced pluripotent stem cell differentiation (Ding et al., 2020, 2021; Ding, 2021). These human MNs naturally expressing heterozygous *TOR1A* mutations revealed impairments in DYT- *TOR1A* neurons in neuronal development, NE morphology, mRNA nuclear export, and protein transport. And found that LMNB1 downregulation can largely ameliorate defects the NE morphology and nuclear transport in patient neurons. These findings indicate that LMNB1 dysregulation may contribute to DYT- *TOR1A* dystonia and provide a novel molecular target for intervention (Sepehrimanesh and Ding, 2020). Compared with healthy MNs, DYT- *TOR1A* MNs have significantly shorter neurites, fewer branches, deformed nuclei, fewer NPCs, and impaired NCT, leading to the mislocalization of mRNA and proteins. This study has important implications for our understanding of the early role of ΔE-TorsinA in neurodevelopment (Sepehrimanesh and Ding, 2020). Therefore, understanding the mechanisms and roles of the synaptic transport pathway involving TorsinA is crucial and can provide new ideas and methods for the prevention and treatment of DYT- *TOR1A* dystonia.

### 4.4. Dopaminergic and cholinergic systems

In the past, dystonia has been considered as a basal ganglia disorder (Bhatia and Marsden, 1994). With the deepening of research in recent years, the role of dysfunction of other brain regions and circuits in dystonia has become increasingly evident (Jinnah et al., 2017). Most researchers currently agree that dystonia is associated with physiological abnormalities in the cortico-ponto-cerebello-thalamo-cortical and cortico-basal ganglia-thalamo-cortical pathways (Poston and Eidelberg, 2012; Jinnah et al., 2017). The basal ganglia circuit model includes a cortico-striatal-pallidothalamic-cortical circuit with primary input into striatum (putamen and caudate) from cortical glutamatergic, thalamostriatal glutamatergic, and nigral dopaminergic projections (Simonyan et al., 2017). The striatum transmits signals to the internal segment of globus pallidus (GPi) and the substantia nigra pars reticulata (SNpr) mainly through two pathways, indirect and direct (Alexander and Crutcher, 1990). Dopaminergic neurons in the substantia nigra pars compacta (SNpc) project to the striatum and synapse with excitatory postsynaptic dopamine D1 receptors of the direct pathway and with inhibitory postsynaptic dopamine D2 receptors on striatal neurons in the indirect pathway touch (Robertson et al., 2017). Accumulating evidence suggests that mutations in *TOR1A, GNAL*, and *ANO3* produce functional changes that affect striatal signal transduction pathways (Herve, 2011; Charlesworth et al., 2012; Goodchild et al., 2013). Interactions between the cholinergic and dopaminergic systems play key regulatory roles in striatal signal transduction. Cholinergic neurons are mainly distributed in the pontine and spinal cord anterior gray matter, releasing acetylcholine as a neurotransmitter to control movement and posture regulation. Dopaminergic neurons are mainly distributed in the midbrain and basal ganglia areas of the brain, releasing dopamine as a neurotransmitter to participate in movement regulation and emotional functions (van den Heuvel et al., 1998; Ichinose et al., 2000; Carbon et al., 2009; Napolitano et al., 2010; Maltese et al., 2017). Under normal circumstances, there is mutual interaction and regulatory relationship between the cholinergic and dopaminergic systems to coordinate and regulate motor control. The effect of the cholinergic system on the dopaminergic system is mainly achieved through the inhibitory effect of cholinergic neurons on dopaminergic neurons. Specifically, cholinergic neurons may promote the activity of dopaminergic neurons by acting on cholinergic receptors on dopaminergic neurons. Conversely, the dopaminergic system may inhibit the activity of cholinergic neurons by acting on D2 receptors (Asanuma et al., 2005; Thony and Blau, 2006; Berman et al., 2013). Mutations in ΔE-TorsinA and DYT- *TOR1A* disease can affect the normal function of the dopaminergic system, leading to motor disorders (Balint et al., 2018). However, recent studies have shown that mutations in ΔE-TorsinA and DYT- *TOR1A* disease may also affect the cholinergic system, further exacerbating motor disorders. Specifically, mutations in ΔE-TorsinA and DYT- *TOR1A* disease may cause excessive activity of cholinergic neurons, thereby exacerbating motor disorders (Carbon et al., 2010; Fox and Alterman, 2015; Balint et al., 2018; Ribot et al., 2019). In addition, mutations in ΔE-TorsinA and DYT- *TOR1A* disease may also affect presynaptic neurotransmitter release, affecting the interaction between the cholinergic and dopaminergic systems. Some studies have shown that there may be an interaction between the cholinergic and dopaminergic systems in presynaptic neurotransmitter release, which may be affected by mutations in ΔE-TorsinA and DYT- *TOR1A* disease, leading to presynaptic neurotransmitter release disorders (Sciamanna et al., 2012a; Liu et al., 2021; Martella et al., 2021; Berryman et al., 2023). Currently, evidence from functional imaging suggests that patients with DYT- *TOR1A* dystonia respond to anticholinergic drugs, and deep brain stimulation (DBS) studies indicate that patients with DYT- *TOR1A* dystonia have functional disorders in both the cholinergic and dopaminergic systems (Carbon et al., 2010; Fox and Alterman, 2015; Balint et al., 2018; Ribot et al., 2019; Sadnicka et al., 2022). The latest DYT- *TOR1A* animal model studies have shown defects in the cell autonomy of dopamine release in the substantia nigra neurons, and the function of striatal projection neurons is also affected, including microstructural changes and cholinergic dysfunction (Page et al., 2010; Song et al., 2013; Maltese et al., 2018; Pappas et al., 2018a; Ponterio et al., 2018). Many studies have reported functional disorders in D2R and have shown that this results from intracellular effects. Research has also found that ΔE-TorsinA can affect the intracellular effects of SPN (spiny projection neurons) (D’Angelo et al., 2017; Zimmerman et al., 2017). These findings help us better understand the impact of TorsinA functional disorders on basal ganglia function and its role in other components of the brain network. In summary, there are complex interactions and regulatory relationships between the cholinergic and dopaminergic systems, which may be affected by mutations in TorsinA and DYT- *TOR1A* disease, leading to motor disorders. Further research will help us better understand the interaction between these neurotransmitter systems, providing guidance for the development of new treatment methods.

### 4.5. Environmental factors

DYT- *TOR1A* dystonia is a genetic disorder caused by a mutation (ΔE mutation) in the *TOR1A* gene. Whereas most research has focused on the genetic mutation and neurobiological mechanisms underlying the disease, an increasing amount of evidence suggests that environmental factors may also influence the onset and severity of DYT- *TOR1A* dystonia. Psychological stress and emotional state are thought to exacerbate the symptoms of DYT- *TOR1A* dystonia. One study found that students with the DYT- *TOR1A* gene mutation reported worsening dystonia symptoms before important exams (Berman et al., 2017). Another study suggested that individuals with dystonia may experience symptom exacerbation under emotional and social stress, indicating that emotional state and psychological stress may affect disease phenotype and severity. Additionally, environmental toxins and chemicals are believed to influence the onset and severity of DYT- *TOR1A* dystonia. Certain chemicals may interfere with the normal function of neurons, which can worsen dystonia symptoms. For example, a study found that some pesticides and pollutants can disrupt the normal function of neurons, exacerbating symptoms of DYT- *TOR1A* dystonia (Rauschenberger et al., 2021). However, it should be noted that the sample sizes in these studies were small, and further research is needed to confirm the exact impact of environmental factors on DYT- *TOR1A* dystonia. Moreover, owing to differences in individual environments and genetics, the impact of environmental factors on individuals may vary. In addition to this, trauma has also been implicated in the development of dystonia. Dystonia accounted for 4.1% of post-traumatic movement disorder cases (Krauss et al., 1996). The Italian Movement Disorders Study Group [2] performed a case control study of 202 dystonia patients and 202 controls matched for age and sex. They found physical trauma as an independent risk factor for the development of dystonia (Defazio et al., 1998, 2017). Overall, current research suggests that environmental factors may influence the manifestation and severity of DYT- *TOR1A* dystonia. Future studies need to further explore these factors and determine their exact role in the onset and development of the disease to provide better prevention and treatment methods.

### 4.6. Molecular convergence with other forms of dystonia

DYT- *TOR1A* dystonia is a hereditary nervous system movement disorder; other hereditary dystonias (such as DYT-*THAP1*, DYT-*SGCE*, and DYT-*ATP1A3*) are also caused by genetic mutations. Although the genetic mutations of these diseases are different, they may affect common pathways, leading to similar clinical manifestations, such as calcium ion homeostasis imbalance, axonal transport disorders, and neuronal cell death. Currently, one of the main areas where the mechanisms of different genetic dystonias overlap is the function and regulation of the ER/NE membrane system stress response. The eukaryotic initiation factor 2α (eIF2α) signaling pathway is an important region where DYT gene molecules aggregate and is associated with ER function. eIF2α is the limiting regulatory subunit of the eIF2 complex. During ER stress, viral infection, or inflammation, cells use their eIF2α kinases to inhibit the translation of unnecessary proteins (Dabo and Meurs, 2012; Bond et al., 2020). The proposal of abnormal activation of the eIF2α pathway has led to the recognition that activation defects of this pathway may be important modifiers of the cellular phenotype of TorsinA dysfunction in cell models, and it has been demonstrated that eIF2α dysfunction is present in fibroblasts of patients with DYT- *TOR1A* dystonia (Beauvais et al., 2016, 2019). eIF2α dysfunction has also been found in DYT-*THAP1*, another form of primary dystonia caused by dominant mutations in the transcription factor *THAP1* (Nayak et al., 2014; Zakirova et al., 2018). Additionally, the DYT-*THAP1* model exhibits similar synaptic plasticity defects to DYT- *TOR1A*, which can be rescued by pharmacological manipulation of eIF2α phosphorylation. EIF2AK2 activity is regulated by interferon-induced double-stranded RNA-dependent protein kinase activator A (*PRKRA*), and a biallelic variant of *PRKRA* is a confirmed cause of dystonia (DYT-*PRKRA*) (DYT-*PRKRA*) (Camargos et al., 2008; Zech et al., 2014). In patient-derived cells carrying the dystonia-causing variant of *PRKRA*, the affinity of *PRKRA-EIF2AK2* interaction is enhanced, and patient fibroblasts exhibit increased and sustained protein kinase R and eIF2α phosphorylation under ER stress (Di Prisco et al., 2014; Trinh et al., 2014; Vaughn et al., 2015; Kuipers et al., 2021). Apart from eIF2α, other translational control pathways may play a role in different forms of dystonia, such as the mTOR pathway, which links energy sensing with protein and lipid metabolism. *GNAL* mutations can cause adult-onset focal or segmental dystonia (DYT-*GNAL*) (Herve, 2011; Alcacer et al., 2012; Fuchs et al., 2013; Pelosi et al., 2017). Altered striatal dopaminergic neurotransmission has been reported in several animal models of monogenic dystonia, such as reduced D2 receptor expression observed in the striatum of patients with both *TOR1A* and *THAP1* variants (Carbon et al., 2009), indicating that dopaminergic signaling abnormalities represent convergent mechanisms downstream of several apparently unrelated genetic defect (Napolitano et al., 2010; Yokoi et al., 2011; Frederick et al., 2019). In addition, defects in striatal postsynaptic dopamine signaling have also been implicated in other hyperkinetic movement disorders, such as levodopa-induced or tardive dyskinesia. Therefore, similar signal integration defects in striatal projecting neurons may be a core feature of hyperkinetic movement disorders beyond inherited dystonia and may share pharmacological targets in the striatum. In addition to these pathways, other pathways may also coexist in different genetic dystonias, such as tyrosine kinase signaling pathways, mitochondrial dysfunction, and nucleosome protein changes. The discovery of these common pathways provides important clues for understanding the pathogenesis of dystonia and helps in the development of more effective treatment strategies.

## 5. Treatment

DYT- *TOR1A* dystonia is an irreversible disease for which there is currently no cure. The goal of treatment is to alleviate symptoms, reduce discomfort, and improve the quality of life for patients. Treatment options include medication, botulinum toxin therapy, surgery, physical therapy, and gene therapy, among other emerging treatments. It is important to note that each treatment has its advantages and disadvantages; therefore, the appropriate treatment plan should be chosen based on the patient’s specific condition. In addition, treatment for DYT- *TOR1A* dystonia is a long-term process that requires collaboration between patients and doctors to regularly monitor and adjust the treatment plan.

### 5.1. Pharmacotherapy

For patients with widespread lesions, oral medication is usually considered for treatment. The drug treatment for DYT- *TOR1A* dystonia mainly targets the symptoms of muscle spasms and stiffness. The following are commonly used drug treatment methods. A) Antispasmodic drugs: the following are some commonly used DYT- *TOR1A* antispasmodic drugs: (1) Non-selective muscarinic acetylcholine receptor antagonist benzhexol (THP) is the preferred oral medication for DYT- *TOR1A* dystonia. THP can alleviate the symptoms of dystonia by reducing the excitability of neuromuscular junctions. It acts on the central nervous system and neuromuscular junctions, inhibiting muscle discomfort and spasms. THP is the only orally administered medication proven to be effective in double-blind placebo-controlled trials (Downs et al., 2021), with an efficacy rate of 71%, which gradually decreases over time. However, most patients cannot use THP because it produces intolerable side effects at the high doses required for treating dystonia. These include dizziness, drowsiness, lack of concentration, dry mouth, constipation, and bradycardia (Jabbari et al., 1989; Schwarz and Bressman, 2009; Thenganatt and Jankovic, 2014; Lumsden et al., 2016). In addition, long-term, prolonged use of THP may lead to drug resistance and dependence, requiring close monitoring and control by doctors. (2) Benzodiazepines: The mechanism of action of these drugs is to enhance the inhibitory effect of GABA (gamma-aminobutyric acid) neurons to suppress the excitability of neuromuscular junctions, thereby reducing the symptoms of muscle tension disorder (Jankovic, 2013). Commonly used drugs include diazepam and clonazepam. Although benzodiazepines can alleviate the symptoms of DYT- *TOR1A* dystonia, there are also some adverse reactions, such as drowsiness, dizziness, lack of concentration, constipation, and dry mouth. They should be used under the guidance of a doctor and monitored for the occurrence of adverse reactions. B) Antidepressants: (1) Selective serotonin reuptake inhibitor drugs, which increase levels of serotonin between neurons by blocking its reuptake, are used to alleviate depression and anxiety disorders. (2) Tricyclic antidepressants such as amitriptyline and imipramine regulate neurotransmitters such as dopamine, norepinephrine, and serotonin to alleviate depression symptoms. C) Antipsychotic drugs: used to treat schizophrenia and other mental illnesses, but in some cases, can also be used to relieve symptoms of DYT- *TOR1A* dystonia. Here are some commonly used antipsychotic drugs for DYT- *TOR1A* dystonia: (1) First-generation antipsychotic drugs such as chlorpromazine and fluphenazine mainly relieve psychotic symptoms by blocking dopamine D2 receptors and reducing symptoms of dystonia. (2) Second-generation antipsychotic drugs such as risperidone and olanzapine: These drugs not only have anti-dopamine effects but also have effects on serotonin and norepinephrine, thereby having a certain relief effect on emotional and dystonia symptoms. D) Sedatives: Sedatives are a class of drugs that can inhibit the central nervous system and mainly produce a relaxing and calming effect on patients. In treating DYT- *TOR1A* dystonia, sedatives can be used to alleviate symptoms such as emotional tension, anxiety, and insomnia, thereby reducing the exacerbation of dystonia. Some commonly used sedatives for DYT- *TOR1A* dystonia are as follows: (1) Benzodiazepines such as lorazepam and alprazolam. These drugs can enhance the effect of GABA neurotransmitters, thereby relieving anxiety and tension symptoms, and also have sedative and hypnotic effects. (2) Barbiturates such as phenobarbital and scopolamine hydrobromide. These drugs mainly produce sedative and anticonvulsant effects by inhibiting the excitability of the central nervous system. They can also alleviate the symptoms of dystonia. It is worth noting that all of the above drugs have certain adverse reactions and should be used under the guidance of a doctor, with strict monitoring of the occurrence of adverse reactions.

### 5.2. Botulinum toxin (BoNT)

The introduction of BoNT in the 1980s represented the most important advancement in the treatment of dystonia (Skogseid, 2014). In a study of patients with isolated dystonia, which included 2,026 positive patients, 61% of patients received BoNT treatment, making it the most commonly used method for treating dystonia to date (Pirio Richardson et al., 2017). BoNT has been used to treat almost all forms of focal and segmental dystonia, as well as many other diseases (Skogseid, 2014). Treatment with BoNT is generally administered by injection directly into the patient’s muscles. A thorough evaluation and screening are required before injection to ensure the patient is suitable for this treatment. The efficacy is generally significant and can significantly alleviate the symptoms of dystonia. The duration of efficacy is generally around 3–6 months, and regular injection maintenance treatment is required. The side effects of BoNT may include injection site pain, muscle weakness, and injection site swelling; however, these side effects are usually short-lived and mild. In rare cases, serious side effects may occur, such as pneumonia or respiratory failure; therefore, treatment should be carried out under the guidance of a doctor. It should be noted that DYT- *TOR1A* BoNT treatment cannot cure dystonia, but only alleviate its symptoms; thus, it needs to be combined with other treatment methods to achieve the best treatment effect. Concurrently, patients need to be regularly reviewed and monitored during treatment to ensure its safety and effectiveness.

### 5.3. Physical therapy

Physical therapy can alleviate symptoms of DYT- *TOR1A* dystonia through various methods, including:

(1) Exercise training: Appropriate exercise training can improve coordination, posture control, and daily activity abilities in patients with dystonia. Exercise training can include balance exercises and flexibility and muscle strength training, among others.
(2) Manual therapy: Manual therapy techniques, such as massage and traction, can relieve muscle tension and pain.
(3) Postural adjustments: Correct postural adjustments can alleviate symptoms, promote blood circulation, and stretch muscles in patients with dystonia.
(4) Functional electrical stimulation: Functional electrical stimulation is a treatment method that stimulates nerves and muscles, increasing muscle activity and control.
(5) Functional training: Functional training is a treatment method that combines different techniques to help patients improve motor skills and movement fluidity and promote muscle coordination and training.

Physical therapy can be part of a comprehensive treatment plan for DYT- *TOR1A* dystonia, helping patients by alleviating symptoms, improving quality of life, promoting recovery, and delaying disease progression. It is necessary to combine individualized assessment and treatment plans for long-term effective treatment and monitoring.

### 5.4. Surgery

Surgical treatment is an optional treatment method for DYT- *TOR1A* dystonia, suitable for patients whose symptoms cannot be controlled by drug therapy or BoNT treatment (Meyer et al., 2017). The following are some common surgical treatment methods for DYT- *TOR1A* dystonia:

DBS: DBS is a surgery that uses implanted electrodes in deep brain nuclei to regulate neuronal activity through electrical stimulation. This surgery can effectively relieve symptoms of dystonia, such as limb tremors and involuntary movements. Common targets in DBS treatment for DYT- *TOR1A* dystonia are the GPi and subthalamic nucleus (Vidailhet et al., 2007; Volkmann et al., 2012). These two targets play an important role in regulating motor function, and regulating neuronal activity in these nuclei through DBS can improve symptoms related to dystonia (Schrader et al., 2011; Volkmann et al., 2014). DBS surgery needs to be performed in a specialized neurosurgery hospital, with relatively high surgical risks, and patients need to undergo a comprehensive evaluation and preoperative preparation. Although DBS can effectively relieve symptoms of dystonia, there are still risks and side effects, such as infection, bleeding, and electrode displacement (Baizabal Carvallo et al., 2012; Ostrem et al., 2017). Therefore, patients need to be treated under a doctor’s supervision and undergo regular postoperative follow-up and adjustment. In addition, DBS needs to be used in combination with other treatment methods to achieve the best treatment effect.

### 5.5. Gene therapy

DYT- *TOR1A* gene engineering refers to the modification or alteration of the DYT- *TOR1A* gene using genetic engineering techniques to treat or prevent the occurrence and development of DYT- *TOR1A* dystonia. This technology includes methods such as gene knockout, gene modification, and gene replacement, aiming to intervene or repair the functional defects of the DYT- *TOR1A* gene to treat or prevent DYT- *TOR1A* dystonia. Currently, DYT- *TOR1A* gene engineering is still in the laboratory research stage and has not been applied in clinical practice. The most common research method is the use of CRISPR-Cas9 technology to knock out the mutation sites in the DYT- *TOR1A* gene, thereby eliminating the impact on DYT- *TOR1A* dystonia (Wu et al., 2022). Researchers have also used gene therapy and other methods to repair the mutation site in the DYT- *TOR1A* gene and restore its normal function (Cruz et al., 2020). However, DYT- *TOR1A* gene engineering still faces many challenges and difficulties, including technical complexity and safety considerations. In addition, since the mutation site of the DYT- *TOR1A* gene is only part of the reason for the disease, even if the mutation site of the DYT- *TOR1A* gene is successfully repaired, it cannot guarantee a complete cure for DYT- *TOR1A* dystonia. In summary, DYT- *TOR1A* gene engineering is a cutting-edge research field that has not yet been applied clinically and requires further research and experimental validation before it can become a safe and effective treatment method.

### 5.6. New emerging treatment methods

In addition to traditional treatment methods such as medication, surgery, and BoNT therapy, there are also some emerging treatment methods being researched and explored for DYT- *TOR1A* dystonia, including (1) optogenetic therapy, which is a gene therapy technique that uses photosensitive proteins and laser light to control neuron activity (Richter et al., 2019; Sciamanna et al., 2020; Schulz et al., 2023); (2) neuroprotective agents, which can protect nerve cells from damage or death, thereby reducing disease symptoms; (3) immunotherapy, which utilizes the immune system to identify and attack diseased cells (Dave and Klein, 2023); and (4) cell therapy, which uses human cells or other types of cells to repair or replace damaged tissues and cells. Although these emerging treatment methods are still in the research and exploration stage, they may bring new breakthroughs and hope for the treatment of DYT- *TOR1A* dystonia (Cruz et al., 2020; Stengel et al., 2020; Akter et al., 2021; Tang et al., 2021). Further research and experimental validation are needed to determine their safety and efficacy.

## 6. Conclusion

In summary, DYT- *TOR1A* dystonia is a rare neurological disorder characterized by involuntary muscle contractions and movement abnormalities. It is caused by a genetic mutation in the *TOR1A* gene, primarily affecting the limbs, neck, and facial muscles. Although the exact mechanism of DYT- *TOR1A* dystonia is not yet fully understood, it is generally believed to involve complex interactions of factors such as genetics, protein deposition, signal transduction, and protein quality control systems. Progress in research has led to a better understanding of the disease and its underlying mechanisms, which may pave the way for more effective treatment and prevention strategies in the future. While there is currently no cure for DYT- *TOR1A* dystonia, a deeper understanding of the disease mechanism provides opportunities for finding treatments. For researchers, the focus is on gaining a deeper understanding of the *TOR1A* gene’s mechanism of action, as well as the relevant neural pathways and protein synthesis and degradation processes. In addition, understanding the impact of this disease on patients’ lives and treatments is critical. In the future, researchers can conduct more clinical trials to explore new treatments, including drug therapy, physical therapy, and neural stem cell therapy.

Future research should prioritize the following:

(1) In-depth understanding of the mechanism of action of the *TOR1A* gene: Although the importance of the *TOR1A* gene is known, there is a need for a deeper understanding of its mechanism of action. Specifically, researchers need to understand how this gene interacts with other genes and proteins and regulates the activity of neurons.
(2) Exploring new treatment methods: Currently, there is no cure for DYT- *TOR1A* dystonia; therefore, we need to explore new treatment methods. Researchers can try to develop new drugs or use new treatments, such as neural stem cell therapy.
(3) Increasing awareness of the disease: Understanding the impact of this disease on patients’ quality of life is essential. Researchers can use methods such as surveys to better understand patients’ quality of life and find better treatment options.
(4) Strengthening international cooperation: DYT- *TOR1A* dystonia is a rare disease that requires global cooperation to better understand its causes and mechanisms. Therefore, strengthening international cooperation will be an important direction for future research.

In summary, future research will further explore the pathogenesis and treatment of DYT- *TOR1A* dystonia, with the hope of providing better treatment and quality of life for patients.

## Author contributions

YF and ZS conceived the idea for the manuscript and contributed to the initial drafting of the manuscript. LZ and LW involved in reviewing and editing the manuscript. All authors read and approved the final manuscript.
